# Gene Ontology Analysis Highlights Biological Processes Influencing Responsiveness to Biological Therapy in Psoriasis

**DOI:** 10.3390/pharmaceutics15082024

**Published:** 2023-07-26

**Authors:** Martina Krušič, Gregor Jezernik, Uroš Potočnik

**Affiliations:** 1 Center for Human Molecular Genetics and Pharmacogenomics, Faculty of Medicine, University of Maribor, Taborska ulica 8, 2000 Maribor, Slovenia; martina.krusic1@um.si (M.K.); gregor.jezernik1@um.si (G.J.); 2 Faculty of Chemistry and Chemical Engineering, University of Maribor, Smetanova ulica 17, 2000 Maribor, Slovenia; 3 Department for Science and Research, University Clinical Centre Maribor, Ljubljanska ulica 5, 2000 Maribor, Slovenia

**Keywords:** psoriasis, biological treatment, therapy outcome, gene ontology

## Abstract

Psoriasis is a chronic, immune-mediated and inflammatory skin disease. Although various biological drugs are available for psoriasis treatment, some patients have poor responses or do not respond to treatment. The aim of the present study was to highlight the molecular mechanism of responsiveness to current biological drugs for psoriasis treatment. To this end, we reviewed previously published articles that reported genes associated with treatment response to biological drugs in psoriasis, and gene ontology analysis was subsequently performed using the Cytoscape platform. Herein, we revealed a statistically significant association between NF-kappaB signaling (*p* value = 3.37 × 10^−9^), regulation of granulocyte macrophage colony-stimulating factor production (*p* value = 6.20 × 10^−6^), glial cell proliferation (*p* value = 2.41 × 10^−5^) and treatment response in psoriatic patients. To the best of our knowledge, we are the first to directly associate glial cells with treatment response. Taken together, our study revealed gene ontology (GO) terms, some of which were previously shown to be implicated in the molecular pathway of psoriasis, as novel GO terms involved in responsiveness in psoriatic disease patients.

## 1. Introduction

Psoriasis is a chronic, immune-mediated, inflammatory skin disease that is characterized by sharply demarcated plaques covered with silver scales or multiple pustules [1]. The prevalence of psoriasis varies by country, and while the numbers are not available for all of them [2], the estimates range from 0.09% to 11.43% on the worldwide scale [3]. This disease can occur in both sexes, with an average age of onset of about 33 years [4]. Psoriasis has a strong genetic background, and to date, more than 80 genetic loci have been associated with its susceptibility [5,6]. There are several subtypes of psoriasis, with plaque psoriasis being the most prevalent [1]. Primary psoriasis affects the skin but can also affect the joints and nails [7]. Moreover, patients with psoriasis have a higher risk of developing comorbidities, including cardiovascular disease, depression, metabolic syndrome, psoriatic arthritis [8], type 2 diabetes and Crohn’s disease [9]. Individuals with mild-to-moderate psoriasis are commonly treated with topical medications, whereas those with moderate to severe psoriasis frequently require systemic therapy [10].

Biologics have been shown to contribute to the successful treatment of psoriasis and improve the quality of life of psoriatic patients. Currently, four classes of biologics are available for psoriasis treatment: inhibitors of TNF-α (adalimumab, etanercept, infliximab and certolizumab pegol), IL-17 (secukinumab, brodalumab and ixekizumab), IL-23 (tildrakizumab, risankizumab and guselkumab) and IL-12/23 (ustekinumab) [11]. The evidence shows that 77–83%, 87–90% and 83–86% of psoriatic patients treated with secukinumab [12], ixekizumab [13] and brodalumab [14], respectively, reached a 75% reduction in the Psoriasis Area Severity Index (PASI) score; thereby, IL-17 inhibitors showed high efficacy. Similarly, guselkumab, an IL-23 inhibitor, has also been shown to be highly effective in treatment for moderate-to-severe psoriasis [15] and has better efficacy in comparison with adalimumab [16]. Moreover, 71% of psoriatic patients treated with adalimumab, an anti-TNF agent, have reached PASI75 [17].

Accumulating knowledge on the molecular pathways that underlie psoriasis has led to a better understanding of the disease’s development and is contributing to the development of promising potential treatment targets [18]. Numerous pharmacogenetic studies have researched the impact of genetic biomarkers on psoriasis disease response, especially to treatment with TNF-α inhibitors [19,20]. On the other hand, fewer pharmacogenetic studies have studied the treatment response to IL-12/23, IL-17 and IL-23 inhibitors [19,20]. It is widely accepted that the response to treatment is heterogeneous, and it is hypothesized that 50–70% of response variation is due to the patients’ genetic background [21]. Since 30–40% of individuals have a poor response or fail to respond to treatment, there is a pressing need for the identification of response biomarkers and their implementation in clinical practice.

A pivotal drawback in psoriasis pharmacogenomic research has been the lack of consensus among the results of different studies. Taking into account the high heterogeneity of responses to biological agents and the observed low replication of biomarkers between studies, the aim of the present study was to pool the existing DNA biomarkers of response and identify their common molecular denominator. To this end, we performed biomarker mining in the published literature, followed by gene ontology (GO) analysis. To better understand the non-responsiveness in psoriasis, we aimed to uncover the molecular architecture that underlies the responsiveness to biological drugs, focusing on anti-TNF and anti-IL12/IL23, by using GO on the set of previously published biomarkers. A similar GO analysis on rheumatoid arthritis, which revealed novel terms in response to anti-TNF therapy, has recently been published by our group [22].

To the best of our knowledge, our study is the first to perform a GO analysis of responsiveness to biological therapy in psoriasis. Our results may contribute to a better understanding of the underlying treatment response/non-response mechanisms.

## 2. Materials and Methods

### 2.1. Literature Search

The study design was based on a workflow described elsewhere [22], with the exception of using some different terms. By using the PubMed database, a literature search was performed in November and December 2022 to identify the studies that investigated the association between psoriasis and treatment response to anti-inflammatory agents. Studies published in the last ten years (November 2011 to November 2022) were considered for inclusion.

Two Mesh search queries that comprised the terms defining the disease, drugs, response, and type of biomarker were defined. Due to the nature of Mesh terms and search queries, we found the initial search query to be insufficient as it left out relevant articles. Consequently, a second search query was implemented to cover the relevant literature fully. The end result is the combination of both search queries.

The first search query:

- Disease terms: “Psoriasis” [Mesh] OR “psoriasis”;
- Drug terms: “Tumor Necrosis Factor Inhibitors” [Mesh] OR “TNF inhibitor” OR (“Etanercept” [Mesh] OR “etanercept biosimilar SB4” [Supplementary Concept]) OR (“Adalimumab” [Mesh] OR “adalimumab biosimilar HS016” [Supplementary Concept]) OR “Certolizumab Pegol” [Mesh] OR (“Infliximab” [Mesh] OR “SB2 infliximab” [Supplementary Concept]) OR “IL-17 inhibitor” OR “brodalumab” [Supplementary Concept] OR “secukinumab” [Supplementary Concept] OR “ixekizumab” [Supplementary Concept] OR “IL-23 inhibitor” OR “tildrakizumab” [Supplementary Concept] OR “risankizumab” [Supplementary Concept] OR “guselkumab” [Supplementary Concept] OR “IL-12/23 inhibitor” OR “Ustekinumab” [Mesh];
- Response terms: “Biomarkers, Pharmacological” [Mesh] OR “Treatment Outcome” [Mesh] OR “Pharmacogenetics” [Mesh] OR “treatment response” OR “response marker” OR “response biomarker” OR “therapy outcome” OR “therapy response”;
- Type of biomarkers terms: “Genetics” [Mesh] OR “Genomics” [Mesh].

The second search query:

- Disease terms: “Psoriasis” [Mesh] OR “Psoriasis/drug therapy” [Mesh] OR “Psoriasis/genetics” [Mesh] OR “Psoriasis/blood” [Mesh];
- Drug terms: “Biological Therapy” [Mesh] OR “Dermatologic Agents” [Mesh] OR “Dermatologic Agents/therapeutic use” [Mesh] OR “Dermatologic Agents/pharmacology” [Mesh] OR “Anti-Inflammatory Agents/therapeutic use” [Mesh] OR “Biological Products/therapeutic use” [Mesh] OR “Antibodies, Monoclonal, Humanized” [Mesh] OR “Antibodies, Monoclonal, Humanized/therapeutic use” [Mesh] OR “Molecular Targeted Therapy” [Mesh] OR “Antibodies, Monoclonal” [Mesh] OR “Antibodies, Monoclonal/therapeutic use” [Mesh] OR “Tumor Necrosis Factor-alpha/antagonists and inhibitors” [Mesh] OR “Etanercept” [Mesh] OR “Etanercept/therapeutic use” [Mesh] OR “Adalimumab” [Mesh] OR “Adalimumab/therapeutic use” [Mesh] OR “Infliximab/therapeutic use” [Mesh] OR “Interleukin-17/antagonists and inhibitors” [Mesh] OR “Interleukin-17” [Mesh] OR “secukinumab” [Supplementary Concept] OR “Interleukin-12/antagonists and inhibitors” [Mesh] OR “Interleukin-23/antagonists and inhibitors” [Mesh] OR “Interleukin-12 Subunit p40/genetics” [Mesh] OR “Ustekinumab” [Mesh] OR “Ustekinumab/therapeutic use” [Mesh];
- Response terms: “Biomarkers, Pharmacological” [Mesh] OR “Treatment Outcome” [Mesh] OR “Biomarkers” [Mesh] OR “Pharmacogenomic Variants” [Mesh] OR “Pharmacogenetics” [Mesh] OR “Biomarkers/metabolism” [Mesh] OR “Genome-Wide Association Study” [Mesh] OR “Precision Medicine” [Mesh];
- Type of biomarkers: “Polymorphism, Genetic” [Mesh] OR “Genetic Markers” [Mesh] OR “Polymorphism, Single Nucleotide” [Mesh] OR “Biomarkers/blood” [Mesh] OR “Genetic Variation/genetics” [Mesh].

*Inclusion/exclusion criteria*. The articles reporting associations between biologics and other types of biomarkers (e.g., RNA, miRNA or protein biomarkers) were excluded from the analysis, since this was not the objective of the present study. Review articles, systematic reviews, duplicate publications, and research published over ten years ago were also excluded from the study. Our search only included articles written in English. The findings of publications reporting single nucleotide polymorphisms (SNPs) and responses (good, poor or nonresponse) to biologics that had statistically significant results (*p* value 0.05) were considered. If studies reported results from univariate or multivariate analysis, significant results from both were included. The genes associated with responsiveness in psoriatic arthritis and studies investigating genetic markers explicitly associated with paradoxical psoriasis were excluded manually.

### 2.2. Gene Ontology Analysis

The gene ontology analysis was carried out using CytoScape [23] with the integrated application ClueGO [24]. The following parameters and selected settings were used to analyze ClueGO:

- Ontology/pathways selected:
  - Biological Process (13 May 2021);
  - Cellular Component (13 May 2021);
  - Molecular Function (13 May 2021);
- Evidence selected: only *All_Experimental*.

After Bonferonni step-down adjustment, a *p* value of less than 5 × 10^−2^ was considered statistically significant.

## 3. Results

### 3.1. Literature Search

In total, 173 studies were found by employing the combination of both of the aforementioned search queries. After removing the duplicate articles, reviews and systematic reviews, 166 publications were reviewed. Based on the inclusion/exclusion criteria described in Section 2 and the initial screening of articles, which included examining the titles and abstracts of the papers, 21 of 166 studies were included for further analysis. Of these 21 studies, 11 examined the response to TNF-α inhibitors, five IL-12/23 inhibitors and five TNF-α and IL-12/23 inhibitors simultaneously. Interestingly, our literature search did not reveal any pharmacogenetic studies that examine the response to IL-17 and/or IL-23 inhibitors in psoriasis. In general, studies did not report differences in responsiveness to biological agents in relation to age, ethnic background or gender.

### 3.2. Biomarkers

Only biomarkers identified in the GO datasets could be processed for gene ontology analysis. Initially, 85 different SNPs were found. Furthermore, the g:Convert Gene ID Converter tool [25] was used to remove potential duplicate biomarkers and to update the biomarker names; finally, 65 genes were included in GO analysis. For SNPs reported with unclear locations (e.g., SNP in *USP8-TNFAIP8L3*), the NCBI Database was used to determine the gene in which the SNP was located. In Table 1, the DNA biomarkers obtained from the examined literature are shown.

### 3.3. Gene Ontology Results

First, we performed a GO analysis of all 65 different associated genes, which revealed 99 unique GO terms, some of which were already known to be implicated in response to biologics. The results with statistically significant hypernyms and GO terms, which have not previously been associated with response in psoriasis, were highlighted and are shown in Table 2. The leading GO term is *regulation of I-kappaB kinase/NF-kappaB signaling* (*p* value = 3.37 × 10^−9^), followed by the GO term *detection of external biotic stimulus* (*p* value = 5.72 × 10^−9^). A statistically significant association with *glial cell proliferation* (*p* value = 2.41 × 10^−5^) was identified.

Furthermore, a comparative GO analysis between two groups was also performed. The genes associated with response to TNF-α inhibitors comprised the first group (cluster 1), and the second group (cluster 2) was represented by the genes involved in response to IL-12/23 inhibitors. Some of the genes associated with the anti-TNF response overlapped with the genes associated with the response to the IL-12/23 inhibitor ustekinumab.

Sixty-four unique GO terms were found by comparative GO analysis. The most interesting results are summarized in Table 3. The results revealed a statistically significant association with the GO term *cellular response to molecule of bacterial origin* (*p* value = 8.06 × 10^−8^), with the term specific for the genes related to anti-TNF response. On the other hand, the analysis indicated an association between the *regulation of interleukin-10 production* (*p* value = 2.58 × 10^−4^) and the genes associated with response to ustekinumab. On the other hand, *positive regulation of interleukin-8* (*p* value = 1.83 × 10^−7^) and *interleukin-6 production* (*p* value = 5.99 × 10^−5^) were not specific for either of the defined clusters.

## 4. Discussion

Genetic biomarkers associated with response to biological therapy are the topic of numerous publications [26,27,28,29,30,31,32,33,34,35,36,37,38,39,40,41,42,43,44,45,46], and their identification could pave the way to personalized medicine [47,48,49]. To the best of our knowledge, we are the first to perform a gene ontology analysis based on the genes previously reported to be associated with response to biologics in psoriasis. Our work highlights the importance of the molecular architecture underlying the response to anti-TNF and anti-IL-12/23 therapy in psoriatic patients.

Our study revealed a statistically significant association between the response to anti-TNF and IL-12/23 in psoriasis and glial cell proliferation. To the best of our knowledge, there is no publication reporting an association between the glial cells and the treatment response to biological drugs in psoriasis. Similarly, there is limited evidence for glial cell involvement in other inflammatory diseases. To date, glial cells have been implicated in inflammatory bowel disease [50,51] and multiple sclerosis [52,53]. Interestingly, in a rat model of germinal matrix hemorrhage, secukinumab has demonstrated a protective role in glial cells [54]. It is known that psoriasis has a range of different symptoms, itching being one of them [55,56]. It is not new that glial cells play an important role in chronic itching and contribute to its exacerbation [57], but the full extent of the involvement of glial cells in psoriasis-induced chronic pruritus has not been clarified yet.

No treatment is currently available for psoriasis-related itching, but clinical trials for its treatment are underway. Ixekizumab and secukinumab, monoclonal antibodies for IL-17 [11], show promising results regarding the attenuation of psoriatic itch. The results of two phase 3 randomized studies [58] have demonstrated a significantly greater improvement in psoriasis-related itch in individuals treated with ixekizumab, compared to those treated with a placebo or etanercept. Two studies have independently shown a reduction in pruritus in subjects who had received secukinumab, in comparison to psoriatic patients who had been treated with a placebo or etanercept [59,60]. In line with this, a study conducted by Blauvelt and colleagues [61] has confirmed that secukinumab leads to greater improvements in alleviating itching than treatment with ustekinumab, an IL-12/23 agent. Furthermore, another IL-17 agent, brodalumab, has been associated with a reduction of itching in moderate-to-severe psoriatic patients [62]. The mechanisms of action of biological drugs responsible for the improvement of psoriatic itch are unknown. Additionally, biological drugs are not the only therapeutics under investigation for relieving the itch in psoriatic subjects. Tofacitinib, an oral Janus kinase inhibitor, has led to rapid improvements in pruritus and has a high potential for treatment of psoriatic itch [63]. Furthermore, Hashimoto and colleagues have detected a decrease in the mRNA expression levels of *IL-22*, *IL-23* and *IL-33* in a mouse model of psoriasis. Interestingly, they have also shown that tofacitinib increases the density of peptidergic epidermal nerve fibers [64]. Altogether, existing evidence underlines the importance of treating the psoriatic itch, thereby improving the quality of life of psoriatic patients.

Interestingly, there is a study addressing the connection between the spinal glial cells and the psoriasis-induced chronic itch in a mouse model [65]. Mice that were treated with imiquimod (IMQ) showed a significant increase in spontaneous scratching after seven days. Thereafter, immunofluorescence was used to measure the expression of the microglial marker IBA-1 and the astrocyte marker glial fibrillary acidic protein (GFAP). The results showed a significantly higher expression of IBA-1 in mice treated with IMQ compared to vehicle-treated mice; however, no significant results were observed with regard to GFAP expression. In addition, the administration of the microglial inhibitor minocycline improved the scratching behavior and decreased the expression of the microglial marker [65].

In order to improve the patients’ quality of life, the underlying molecular mechanisms associated with itching have been researched by several groups. The current evidence reports an association between lipocalin-2 (LCN-2) and itching in psoriatic subjects. LCN-2 is a glycoprotein whose expression is up-regulated in adipose tissue [66], chronic kidney disease [67], human bone marrow cells [68] and heart disease [69,70]. Interestingly, a few studies have shown that increased LCN-2 levels have an important role in psoriasis [71,72,73] and that LCN-2 is secreted by neutrophils and keratinocytes [73,74]. Moreover, neuronal production of LCN-2 has been shown to be an additional signal for glial activation [75], thereby further confirming the glial cell proliferation finding of the present study. Additionally, LCN-2 has multiple roles in the regulation of astrocytes [76], which strengthens its connection to the observed GO term. It has also been suggested that LCN-2 may up-regulate the GFAP protein [76], thus further strengthening the interconnection of LCN-2 to the discovered GO term.

Aizawa and colleagues [71] have investigated serum samples obtained from psoriatic patients, patients with atopic dermatitis and healthy controls. The degree of itching was measured with a visual analog scale (VAS), which is an evaluated method for pruritus assessment [77], and LCN-2 protein levels were determined by ELISA. They reported that individuals with psoriasis had a significantly higher serum LCN-2 concentration compared to healthy controls. When the serum LCN-2 levels of patients with and without itch were compared, significantly higher levels were found in the group with itch. In addition, pre- and post-treatment serum LCN-2 levels were compared in psoriatic patients treated with TNF-α (adalimumab, infliximab) and IL-17 (secukinumab, brodalumab) inhibitors. Treatment with biologics was shown to decrease serum LCN-2 concentrations in psoriasis patients with itch [71]. Similarly, another group of researchers has reported a high expression of LCN-2 in the skin layers of psoriatic patients [73]. The experiment using the IMQ-induced psoriasis mouse model showed a reduction in epidermal thickness and a reduced number of infiltrated inflammatory cells (which play a key role in the pathogenesis of psoriasis [1]) in the dermis [73]. Collectively, the reported findings have shown that biologics contribute to the alleviation of psoriatic-related itch. Moreover, considering that the reduction of serum LCN-2 levels has been shown to improve pruritus, LCN-2 could be a potential clinical biomarker for itch in psoriasis.

Furthermore, the NF-kappaB and JAK/STAT pathways play crucial roles in the molecular pathway of psoriasis [78,79], and NF-kB is a significant player in the regulation of inflammation [80]. The results of the present study have uncovered an association between the treatment response and the regulation of NF-kappaB signaling as one of the most significant GO terms. Currently, studies of NF-kB and treatment response in psoriasis are scarce. A study published by Andres-Ejarque and colleagues [81] has highlighted the importance of NF-κB signaling in response to adalimumab in psoriasis. Patients with enhanced NF-κB signaling, which causes an increased maturation phenotype in dendritic cells (DC) and an increased fraction of IL-17^+^ T cells in the blood and skin before initiation of therapy, have shown an inferior efficiency of the adalimumab response [81]. In addition, the aforementioned discovery indicates that there is a specific disease endotype that directly influences the clinical response to adalimumab [81]. However, due to the limitations of our methods, we are unable to determine if this result is truly a mechanism of non-response or a consequence of the responder/non-responder definition, i.e., non-responders have more uncontrolled symptoms and thus greater inflammation than responders.

Furthermore, our results have highlighted granulocyte-macrophage colony-stimulating factor (GM-CSF) production regulation in response to biologicals in psoriasis. Comparative GO analysis omits this result when anti-TNF and anti-IL-12/23 response biomarker lists are split, suggesting it is common to both treatments and that it only appears when all biomarkers are present for sufficient statistical power. Over three decades ago, elevated expression of GM-CSF was detected in psoriatic lesions [82] and later also in psoriasis patients’ sera as well as skin lesions [83]. Keratinocytes are known to produce GM-CSF [84], which has thus been investigated as a therapeutic target [85]. Targeting GM-CSF would limit keratinocyte proliferation and infiltration of the immune cells, which would in turn indirectly affect the Th17 cell pathway [86,87]. However, namilumab, a monoclonal antibody targeting GM-CSF, has failed to produce a significant therapeutic effect in psoriasis patients [88]. Nevertheless, GM-CSF has previously been reported as a response biomarker for both anti-TNF and anti-IL-12/23 treatment in psoriasis [89]. Solberg and colleagues have reported that approximately a fifth of psoriasis patients have detectable GM-CSF levels in psoriatic lesions [89]. In patients with detectable pre-treatment GM-CSF levels, the fluctuations of pre-treatment GM-CSF levels correlated with the variability of treatment response to both anti-TNF and anti-IL-12/23 treatments [89]. This further suggests that patients with detectable pre-treatment GM-CSF levels may be an important subgroup for anti-TNF and anti-IL-12/23 treatment. The notion that GM-CSF may serve as both an anti-TNF and anti-IL-12/23 biomarker is in line with our GO results.

Interestingly, the gene *ADRA2A* is shared between the GO terms GO:0050995 and GO:0051043. To date, only the SNP rs553668 in the *ADRA2A* gene has been associated with anti-TNF treatment response in psoriasis [36]. However, it remains unclear what role *ADRA2A* plays in the anti-TNF response in psoriasis or psoriasis pathogenesis. *ADRA2A* is known to play a role in platelet aggregation [90], which constitutes a part of a greater network of inflammatory processes [91]. Overall, *ADRA2A* may be involved in inflammation by affecting platelets, but further functional analyses are required.

The chronic inflammation in psoriasis is a result of crosstalk between different cells. Moreover, cytokines also play an important role in the pathogenesis of psoriatic disease [92]. The IL-1 family is crucial in psoriasis pathogenesis [93]. This group includes IL-1β, which has an important role in IL-17-producing T cell differentiation and activation [94]. Lu and colleagues [95] have examined the mRNA and protein levels of IL-1β in response to treatment with guselkumab in psoriatic patients. Their findings suggest that both mRNA and protein levels of IL-1β are associated with response to the IL-23 inhibitor guselkumab [95]. Interestingly, it has been shown that expression of IL-1β was down-regulated in fast responders compared to slow responders (whose expression was elevated) [95]. Therefore, IL-1β has been suggested to be associated with response to guselkumab in psoriatic patients. On the other hand, a study published by Liu and colleagues [96] examined the correlation of inflammatory cytokine levels in response to etanercept. Serum levels of several inflammatory cytokines, including IL-1β, were decreased six months after treatment initiation with etanercept (*p* value < 0.001) [96]. Moreover, statistical significance was observed in serum levels of IL-1β, which were increased in responders in comparison to non-responders (response was measured as PASI75; *p* value = 0.007) [96].

Although the strength of our study is the well-defined inclusion/exclusion criteria and the carefully reviewed literature, it is possible that some patients with paradoxical psoriasis are also included in the study, which represents a potential limitation of the study. The advent of additional psoriatic symptoms in psoriasis patients after therapy initiation can be considered a non-response to the biological drug, as worsening symptoms would result in a lower PASI score. As such, paradoxical psoriasis, which can be clinically difficult to distinguish from existing psoriasis, may be considered a manifestation of biological drug non-response. Thus, studies reporting non-response biomarkers in psoriasis may have had patients with paradoxical psoriasis defined as non-responders.

## 5. Conclusions

Although a considerable number of studies have investigated the response to biologics in patients with psoriasis, the identified genes are not commonly replicated in other/validation studies, with the exception of *HLA-C*. There is also a notable lack of studies investigating the response to treatment with IL-17 and IL-23 inhibitors, despite evidence of a good treatment response to these therapeutics. The main limitation of our study was the relatively small number of articles included in the analysis and the inclusion of articles reporting only genetic markers. Furthermore, subset analysis by age, gender, race, etc. has been impeded by the limited clinical data. Taken together, our results suggest that glial cells may contribute to the treatment response in psoriatic patients treated with biologics, but additional studies are warranted to further explore our findings. Glial cells have been known to be associated with the development and maintenance of itch, one of the symptoms present in psoriasis. Previously published publications have reported that LCN-2 may be a useful clinical biomarker of psoriatic-related itch treatment. Finally, our findings have highlighted the importance of understanding the molecular background of treatment responses. This understanding can lead to more effective treatments by unraveling novel drug targets (e.g., the aforementioned LCN-2). Furthermore, a deeper genetic investigation of key pathways, such as those involving glial cells, could lead to pre-treatment stratification of patients or genetic testing to aid in clinical decision making when selecting the first biological therapy. Consequently, genetic testing and novel pharmaceutical developments undoubtedly hold great potential to improve patients’ quality of life.

## Figures and Tables

**Table 1:** Genes associated with psoriasis response to biological drugs.

Associated Gene	Biological Treatment	Study
*AKAP13*	TNF-α inhibitors	[26]
*SUPT3H*		
*CDH12*		
*HNRNPKP3*		
*NPFFR2*		
*IL12B*	TNF-α inhibitors	[27]
*NFKBIZ*	TNF-α inhibitors	[28]
*HLA-C* (*Cw6*) *		
*HLA-C* (*HLA-C* * *06:02*) *	TNF-α inhibitors,	[29]
	IL-12/23 inhibitors	
*IL10*	TNF-α inhibitors	[30]
*IVL*		
*PGLYRP3*		
*SPRR2F*		
*TNFR1*		
*MYD88*		
*IL12B*		
*PTTG1*		
*SLC22A4*		
*CDKAL1*		
*TNFAIP3*		
*NFKBIA*		
*AP4E1* (*USP8-TNFAIP8L3*) *		
*MAP3K14*		
*ZNF816A*		
*SLC9A8*		
*HLA-C* (*HLA-Cw0602*) *	IL-12/23 inhibitors	[31]
*IL1B*	TNF-α inhibitors	[32]
*LY96*		
*TLR2*		
*IL19*	TNF-α inhibitors	[33]
*PTGS2*		
*CTNNA2*		
*LINC01185* ^‡^		
*PTTG1*		
*HLA-B* (*HLAB/MICA*) *		
*C17orf51* ^‡^		
*MAP3K1*		
*ZNF816* (*ZNF816A*) *		
*SDC4*		
*GBP6*		
*IVL*		
*IL12B*		
*HTR2A* (*5-HTR2A*) *		
*PSTPIP1*		
*HLA-C* (*HLA-C* * *06*) *	IL-12/23 inhibitors	[34]
*TNFRSF1A*	IL-12/23 inhibitors	[35]
*HTR2A*		
*NFKBIA*		
*ADAM33*		
*IL13*		
*CHUK*		
*C17orf51* ^‡^		
*ZNF816* (*ZNF816A*) *		
*STAT4*		
*SLC22A4*		
*C9orf72*		
*SPEN*	TNF-α inhibitors	[36]
*JAG2*		
*MACC1*		
*GUCY1B3*		
*PDE6A*		
*CDH23*		
*SHOC2*		
*LOC728724* ^‡^		
*ADRA2A*		
*KCNIP1*		
*IL23R*	TNF-α inhibitors	[37]
*PGLYRP4* (*PGLYRP4-24*) *		
*PGLYRP3* (*PGLYRP3-19*) *		
*PGLYRP4* (*PGLYRP4-16*) *		
*PGLYRP4* (*PGLYRP4-07*) *		
*PGLYRP4* (*PGLYRP4-30*) *		
*PTGS2*		
*IL19*		
*LCE*		
*TNFR1*		
*C17orf51* ^‡^		
*ZNF816 (ZNF816A)* *		
*CTNNA2*		
*REL*		
*MYD88*		
*IL12B*		
*PTTG1*		
*MAP3K1*		
*TSBP1* (*C6orf10*) *		
*HLA-C*		
*CD84*	TNF-α inhibitors,	[38]
*IL12B*	IL-12/23 inhibitors	
*TNFAIP3*		
*FCGR3A*	TNF-α inhibitors	[39]
*HLA-C*	TNF-α inhibitors,	[40]
*TRAF3IP2*		
*TNFAIP3*		
*HLA-A*	IL-12/23 inhibitors	
*ERAP1*		
*HLA-C (HLA-Cw6)* *	IL-12/23 inhibitors	[41]
*IL12B*		
*IL6*		
*IL17F*	TNF-α inhibitors,	[42]
	IL-12/23 inhibitors	
*TNFRSF1B*	TNF-α inhibitors,	[43]
	IL-12/23 inhibitors	
*SLOCO1C1* (*PDE3A-SLCO1C1*) *	TNF-α inhibitors	[44]
*HLA-C* (*HLA-Cw6*) *	IL-12/23 inhibitors	[45]
*TNF (TNF-α)* *	TNF-α inhibitors	[46]
*IL23R*		
*HLA-C (HLA-Cw6)* *		

* Gene names that were reported in the studies are depicted in brackets. ^‡^ Genes that were excluded from analysis due to the lack of g:Profiler data and/or due to the lack of data within the Gene Ontology Consortium database (e.g., non-coding sequences).

**Table 2:** Significant GO terms associated with psoriasis response to biological drugs.

GO ID	GO Term	Term *p* Value	Term *p* Value Corrected with Bonferroni Step Down	Associated Genes Found
GO:0043122	regulation of I-kappaB kinase/NF-kappaB signaling	4.32 × 10^−11^	3.37 × 10^−9^	[AKAP13, CHUK, IL1B, MAP3K14, MYD88, TIRAP, TNF, TNFAIP3, TNFRSF1A, TRAF3IP2]
GO:0098581	detection of external biotic stimulus	7.43 × 10^−11^	5.72 × 10^−9^	[HLA-A, HLA-B, LY96, PGLYRP3, PGLYRP4, TLR2]
GO:0051043	regulation of membrane protein ectodomain proteolysis	2.67 × 10^−9^	1.90 × 10^−7^	[ADRA2A, IL10, IL1B, TNF, TNFRSF1B]
GO:0032645	regulation of granulocyte macrophage colony-stimulating factor production	9.54 × 10^−8^	6.20 × 10^−6^	[CD84, IL12B, IL17F, IL1B]
GO:0060252	positive regulation of glial cell proliferation	4.09 × 10^−7^	2.41 × 10^−5^	[IL1B, IL6, TNF]
GO:0032651	regulation of interleukin-1 beta production	1.93 × 10^−6^	1.02 × 10^−4^	[IL17F, IL6, MYD88, TNF, TNFAIP3]
GO:0045672	positive regulation of osteoclast differentiation	4.84 × 10^−6^	2.23 × 10^−4^	[IL12B, IL17F, TNF]
GO:0050769	positive regulation of neurogenesis	7.40 × 10^−6^	3.11 × 10^−4^	[IL1B, IL6, SPEN, TNF]
GO:0050995	negative regulation of lipid catabolic process	8.84 × 10^−6^	3.45 × 10^−4^	[ADRA2A, IL1B, TNF]
GO:0050796	regulation of insulin secretion	2.37 × 10^−5^	6.87 × 10^−4^	[ADRA2A, IL1B, IL6, TNF]

**Table 3:** Comparative GO analysis. “Cluster 1” refers to anti-TNF treatment biomarkers while “Cluster 2” refers to anti-IL-12/23 treatment biomarkers.

GO ID	GO Term	Term *p* Value	Term *p* Value Corrected with Bonferroni Step Down	Cluster	Genes Cluster 1	Genes Cluster 2
GO:0071219	cellular response to molecule of bacterial origin	1.47 × 10^−9^	8.06 × 10^−8^	Specific for Cluster 1	[IL10, IL1B, LY96, TLR2, TNF, TNFAIP3, TNFRSF1B]	[IL1B, IL6, TNFAIP3]
GO:0043123 *	positive regulation of I-kappaB kinase/NF-kappaB signaling	1.72 × 10^−9^	9.31 × 10^−8^	No Specific Cluster	[AKAP13, MAP3K14, MYD88, TNF, TNFRSF1A, TRAF3IP2]	[CHUK, TIRAP, TNFRSF1A]
GO:0051043 *	regulation of membrane protein ectodomain proteolysis	2.43 × 10^−9^	1.29 × 10^−7^	Specific for Cluster 1	[ADRA2A, IL10, IL1B, TNF, TNFRSF1B]	[IL1B]
GO:0032757	positive regulation of interleukin-8 production	3.59 × 10^−9^	1.83 × 10^−7^	No Specific Cluster	[IL1B, MYD88, TLR2, TNF]	[IL1B, IL6, TIRAP, TLR5]
GO:0032755	positive regulation of interleukin-6 production	1.62 × 10^−6^	5.99 × 10^−5^	No Specific Cluster	[IL1B, TLR2, TNF]	[IL1B, IL6, TIRAP]
GO:0050995 *	negative regulation of lipid catabolic process	8.37 × 10^−6^	2.43 × 10^−4^	Specific for Cluster 1	[ADRA2A, IL1B, TNF]	[IL1B]
GO:0032653	regulation of interleukin-10 production	9.54 × 10^−6^	2.58 × 10^−4^	Specific for Cluster 2	[IL12B, IL23R]	[IL12B, IL13, IL6]

* Terms obtained in comparative analysis that overlap with the results of the first analysis.

## Data Availability

Data are contained within the article or the Supplementary Materials (Supplementary Materials File S1).

## Supplementary Materials

The following supporting information can be downloaded at: https://www.mdpi.com/article/10.3390/pharmaceutics15082024/s1.
